# Cardiovascular magnetic resonance in the evaluation of heart valve disease

**DOI:** 10.1186/s12880-017-0238-0

**Published:** 2017-12-29

**Authors:** G. S. Gulsin, A. Singh, G. P. McCann

**Affiliations:** Department of Cardiovascular Sciences, University of Leicester and the NIHR Biomedical Research Centre, Glenfield Hospital, Groby Road, Leicester, UK

## Abstract

**Background:**

Over the last 25 years, cardiovascular magnetic resonance imaging (CMR) has emerged as an alternative to echocardiography for assessment of valvular heart disease (VHD). Although echo remains the first-line imaging modality for the assessment of patients with VHD, CMR can now provide a comprehensive assessment in many instances. Using a combination of techniques, CMR provides information on valve anatomy and enables quantitative analysis of the severity of the valve lesion.

**Main text:**

In this review, the fundamentals of CMR in assessment of VHD are described, together with its strengths and weaknesses. We detail the utility of CMR for studying all aspects of VHD, including valve anatomy, flow quantification as well as ventricular volumes and function. The optimisation of CMR for evaluating the commonest valve lesions (aortic stenosis, aortic regurgitation, mitral regurgitation, mitral stenosis) as well as in right-sided VHD and prosthetic valves is summarised. The focus of this review is to enable the reader to optimise the use of CMR in his or her own evaluation of heart valve lesions in clinical practice.

**Conclusions:**

CMR can be used for the comprehensive evaluation of VHD. This exciting, non-invasive imaging modality is likely to have increasing utility in the clinical evaluation of patients with VHD.

## Background

Over the last 25 years, CMR has emerged as an alternative to echocardiography for assessment of valvular heart disease (VHD). Although echocardiography remains the first-line imaging modality for the assessment of patients with VHD, CMR can now provide a comprehensive assessment in many instances. This is especially true in patients with poor acoustic windows and where echocardiography is limited by operator dependence.

Using a combination of techniques, CMR provides information on valve anatomy and enables quantitative analysis of the severity of the valve lesion. CMR allows unparalleled evaluation of the consequences of valve disease on the relevant ventricle and on the anatomy of surrounding structures. Tissue characterisation, particularly with late gadolinium enhanced (LGE) also provides additional information regarding myocardial infarction or fibrosis, which may be clinically relevant in patients with VHD. The relative strengths and weaknesses of CMR in the evaluation of VHD are listed in Table 1, below.

**Table 1:** The strengths and weakness of CMR in the evaluation of VHD

CMR strengths	CMR weaknesses
Unlimited windows	Regurgitant jet visualisation inferior to echocardiography
Excellent image quality	Low through-plane spatial resolution
Flow quantification	Low(er) temporal resolution
Gold standard imaging modality for left and right ventricle assessment	Averages of multiple R-R intervals
Multi-parametric comprehensive assessment (LGE, T1 mapping, ischaemia)	Peak velocities can be underestimated
	Flow quantification can be prone to errors

In this article, we review the role of CMR in the evaluation of VHD, with emphasis on clinical applications of CMR techniques. In particular, we focus on the four key left-sided valve pathologies: aortic stenosis (AS), aortic regurgitation (AR), mitral stenosis (MS) and mitral regurgitation (MR). We also highlight the role of CMR in the assessment of other, rarer valve lesions.

## General principles

### CMR pulse sequences for evaluation of valvular heart disease

Several CMR radiofrequency pulse sequences are used in the assessment of VHD. The pulse sequences have utility in different circumstances applicable to VHD (Table 2).

**Table 2:** CMR pulse sequences with utility in the evaluation of VHD [4]

CMR pulse sequence	Utility in VHD
Steady-state free precession	Valve anatomy and motion Ventricular volumes and function Turbulent blood flow jet visualisation
Gradient echo	Valve anatomy and motion Turbulent blood flow jet visualisation Prosthetic valve assessment
Phase contrast	Flow velocity Forward and reverse volumes
Turbo spin echo	Evaluation of valve masses
Segmented inversion recovery gradient echo	Evaluation of valve masses

### Heart valve anatomy

The complete valve anatomy can be visualised by CMR, including the valve leaflets, chordae tendinae and papillary muscles. CMR can also identify the presence of valve masses, such as vegetations, thrombi and tumours, highlighting their attachment site and mobility [1, 2].

The steady-state free precession (SSFP) pulse sequence is the most widely used for assessment of valve morphology and function [3]. To visualise each valve throughout systole and diastole, image acquisition is gated to the ECG over several cardiac cycles. Each slice can be obtained within a single breath hold lasting only 5-8 s. SSFP sequences are favoured for their high contrast between blood pool and surrounding structures, together with a high signal-to-noise ratio. SSFP can be used to produce 2D cine images of all four heart valves in any prescribed plane, with multiple phases throughout the cardiac cycle. This allows all four valves to be imaged irrespective of challenging thoracic or cardiac anatomy and is particularly useful for right-sided heart valves, which are often difficult to study with echocardiography. Furthermore, planimetry enables direct measurement of valve orifice area for stenotic valves.

**Fig. 1:**
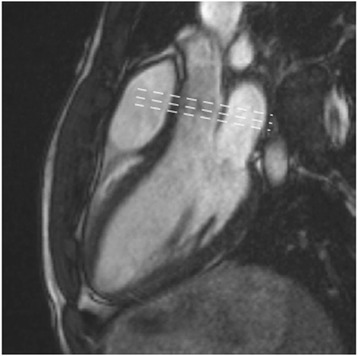
SSFP cine three-chamber image of the normal aortic valve. Dashed lines indicate location of multiple parallel slices that should be used to avoid errors in planimetry. Source: University Hospitals of Leicester NHS Trust

Several limitations of CMR assessment of valve anatomy exist. Foremost is the relatively large slice thickness (typically 5-8 mm) of CMR cine images. Thin structures as cardiac valves (which are usually 1-2 mm thick) are therefore susceptible to partial volume effects. Careful planning of imaging slices perpendicular to the valve enhance assessment and in plane resolution will typically be in the order of 1.0–1.5 mm if a matrix of 256 is used and the field of view is 260mm x 390mm. Thinner slice thickness (4–5 mm) can improve the accuracy of evaluation but at the expense of reduced signal to noise ratio. This is particularly important for measurement of valve orifice area, where positioning of the slice image at the valve tips is vital to avoid error in planimetry. Such errors can be avoided by imaging across the valve area with multiple parallel slices to determine the position closest to the valve tips (Fig. 1).

### Flow

SSFP and gradient echo cine images provide visualisation of turbulent flow jets across stenosed or regurgitant valves. These are seen as signal voids and occur due to spin-dephasing in moving protons. A visual assessment of the site and direction of stenotic or regurgitant flow, similar to that with colour flow echo Doppler, can be made prior to further evaluation of the valve lesion. In this regard SSFP is less sensitive than gradient echo in depicting regurgitant jets. In gradient echo sequences the sensitivity for detecting spin-dephasing is a function of the echo time, i.e. the longer the echo time, the larger and more pronounced the jet [4].

Flow velocity can be directly quantified by CMR using through-plane phase contrast velocity mapping. Phase-contrast pulse sequences are based on the property that protons moving within a magnetic field gradient acquire a shift in the phase of their rotational spin compared with stationary protons. The magnitude of this shift is proportional to velocity. The net phase of moving protons is proportional to the velocity of blood and can be displayed as a phase map, where different velocities are represented by different signal intensities. Flow in the direction of the phase-encoding appears white whereas flow in the opposite direction appears black. Stationary objects (i.e. those with a phase-shift of zero) appear grey.

**Fig. 2:**
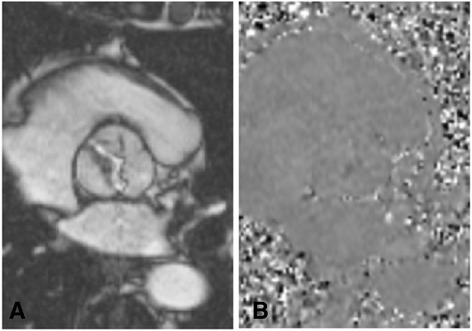
**a** magnitude and **b** phase images generated by CMR flow velocity mapping in a patient with a type 1 bicuspid aortic valve. The magnitude image provides visualisation of the valve anatomy and the phase map is used to calculate velocity within each pixel. Source: University Hospitals of Leicester NHS Trust

**Fig. 3:**
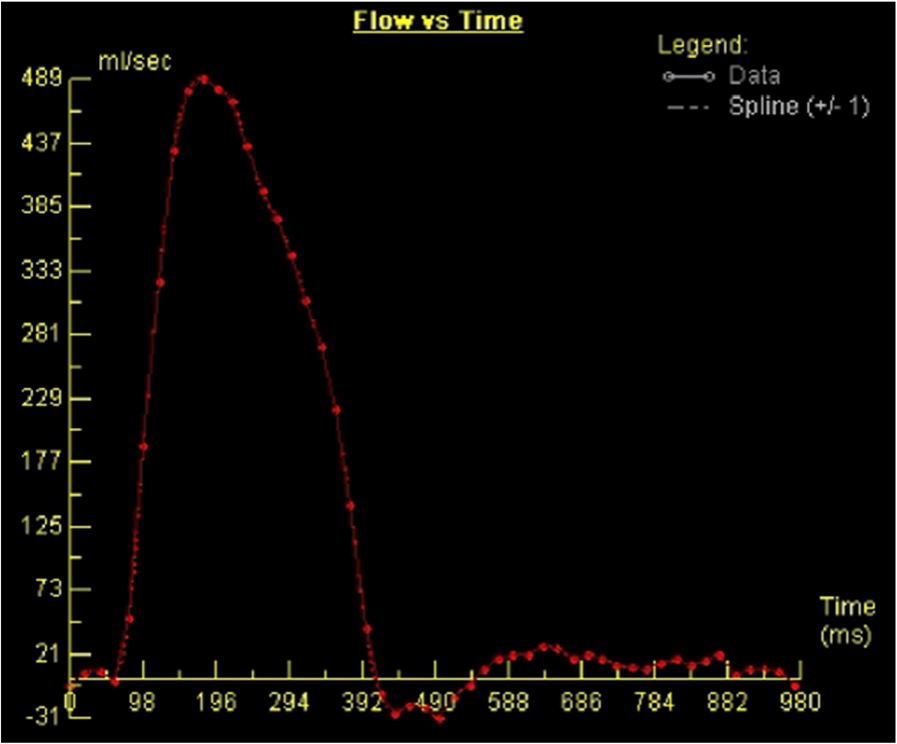
A normal ascending aortic flow-time graph generated by CMR through-plane phase-contrast velocity mapping. Source: University Hospitals of Leicester NHS Trust

Velocity mapping generates two sets of images: 1) magnitude images, which delineate the anatomy of the vessel(s) being studied and their surrounding structures, and 2) phase velocity maps, where the velocities within each pixel are encoded (Fig. 2). The region of interest is traced on these images for each frame of the cardiac cycle. Flow volume (cm^3^/s) is calculated by multiplying the velocity within each pixel (cm/s) by the area (cm^2^) and a flow-time graph can be generated over one cardiac cycle (Fig. 3).

CMR 2D phase-contrast flow measurements can be performed with free-breathing or breath-held techniques. Breath-held acquisitions are shorter, but may not account for the physiological effects of breathing on cardiac filling. Breath holding may also prove difficult in patients with dyspnoea. Free-breathing techniques have a longer sampling time and require temporal averaging of flow measurements, but may better account for physiological effects of breath holding. Because phase-contrast flow mapping relies on ECG gating to average flow information over multiple cardiac cycles, it is prone to errors in patients with arrhythmias, where there are beat-to-beat flow variations. In such cases, real-time phase contrast flow imaging may be performed without ECG gating [5].

CMR flow measurements correlate strongly with Doppler and invasive in-vivo flow measurements [6–8]. However the temporal resolution of CMR flow measurement (25–45 ms) is lower than that of continuous wave Doppler echo (2 ms). For high flow velocities of short duration, there is the risk that CMR flow measurement may underestimate peak velocity. Nevertheless the temporal resolution of CMR is sufficient for most flow measurements.

A key limitation of clinical flow acquisition is the occurrence of positive or negative phase offset errors, which occur due to local non-compensated eddy currents. Phase offset errors can lead to considerable miscalculations in flow quantification. Even small velocity offset errors can lead to sizeable flow quantification errors, because flow volume is calculated by integrating velocities across the cross-sectional area of the vessel over the entire cardiac cycle [9]. Velocity offset errors can be minimised by ensuring that the vessel of interest is positioned into the isocenter plane for flow imaging. Post-acquisition offset correction methods may also improve the reliability of flow quantification. In routine practice, clinicians should be aware of potential inaccuracies in flow quantification and aim to internally validate flow measurement in the aorta versus LV stroke volumes in patients without mitral regurgitation. Alternatively, scanning a stationary gel phantom with identical flow acquisitions as a baseline reference for zero velocity is proposed as a measure to tackle phase offset correction [10].

### Ventricular volumes and function

**Fig. 4:**
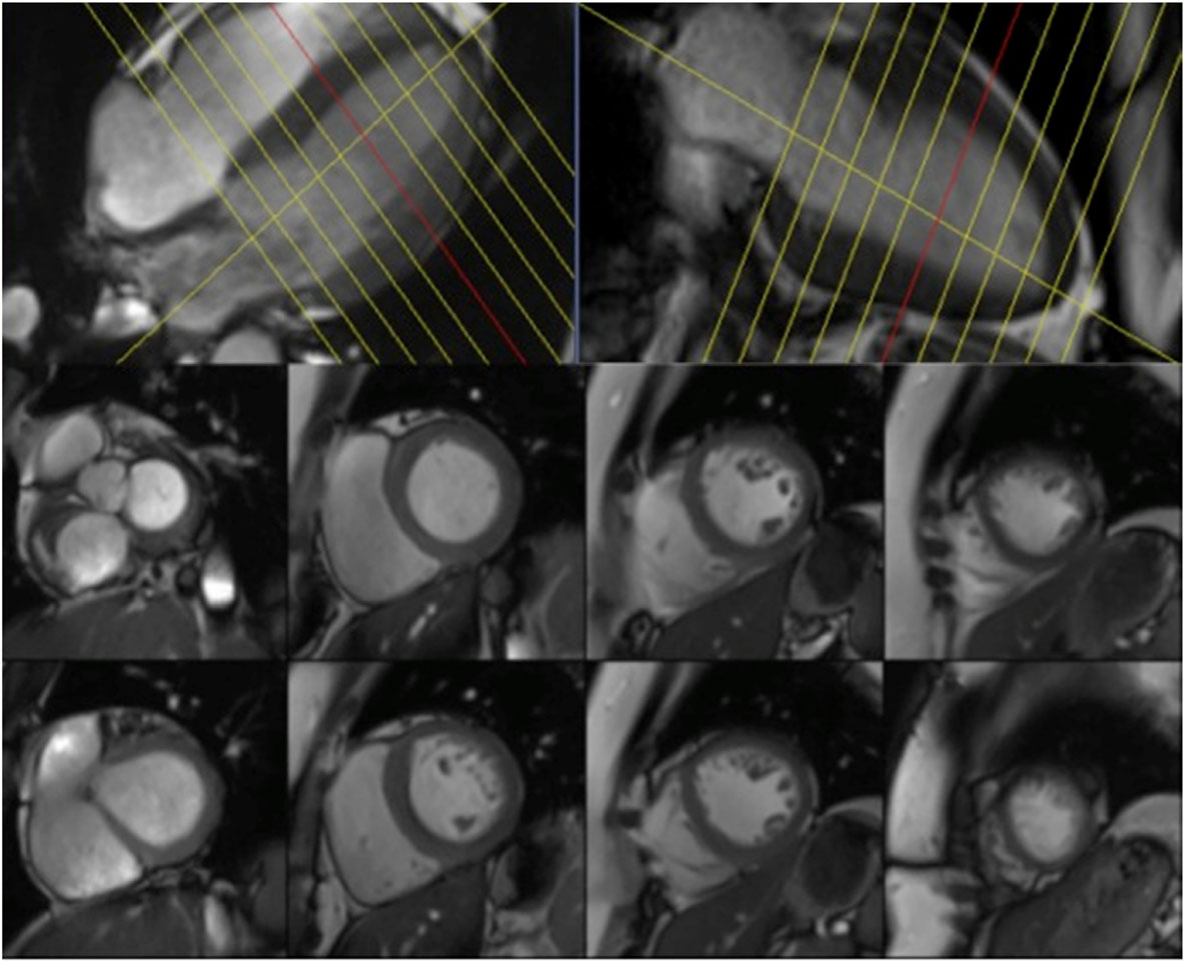
An example of the planning used for the short-axis SSFP cine stack shown on 4- chamber and 2-chamber slices (top panel), with examples of some short-axis slices (bottom panel). Source: University Hospitals of Leicester NHS Trust

**Fig. 5:**
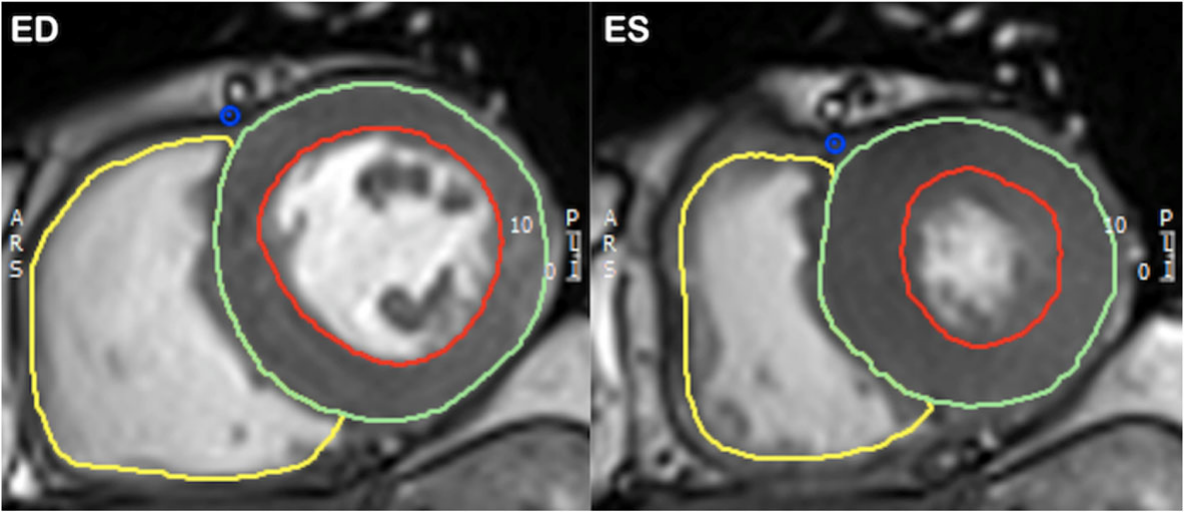
An example of epicardial (green), endocardial (red) and right ventricular (yellow) contours at end-diastole (ED) and end-systole (ES). Source: University Hospitals of Leicester NHS Trust

CMR is the gold-standard imaging modality for evaluation of left and right ventricular volumes, mass and function [11]. Accurate assessment of the consequence of a valve lesion on the relevant ventricle is imperative in establishing the timing for intervention. Following acquisition of the localiser images, cine imaging is acquired, using the SSFP pulse sequence, in the two-, three- and four- chamber views. The long-axis views are then used to plan a full “stack” of short-axis slices, the first being planned at the mitral valve annulus, perpendicular to the inter-ventricular septum, with a slice every 10 mm, until full coverage of the left ventricle (LV) is achieved (Fig. 4). For quantification, LV epicardial and endocardial contours are drawn at end-diastole and end-systole (Fig. 5), allowing calculation of LV end-diastolic volume (LVEDV), LV end-systolic volume (LVESV), stroke volume (SV), LV ejection fraction (LVEF) and LV mass (LVM) [12].

### Aortic stenosis (AS)

AS is the commonest valve disease requiring surgery in the developed world. Up to 3% of individuals aged ≥75 years are affected by AS, most commonly occurring due to calcific degeneration of the aortic valve [13, 14]. Other causes of AS include a congenital bicuspid aortic valve or rheumatic valve disease [14]. Calcific AS advances from a prolonged asymptomatic period with progressive narrowing of the aortic valve orifice. There is a corresponding increase in the pressure gradient across the aortic valve with associated LV pressure overload and LV hypertrophy. Subsequent onset of symptoms – typically angina, heart failure and syncope – portends a poor prognosis without intervention, with death usually occurring within 5 years [15]. Most patients with asymptomatic disease are recommended to undergo periodic monitoring to assess severity and adverse ventricular remodelling [16].

Application of CMR in the evaluation of AS includes anatomical assessment of the aorta and aortic valve, quantification of LV volumes, mass and function, and calculation of stenotic jet velocity [3]. The three standard measures used to establish the severity of AS are valve area, peak velocity and pressure gradient (Table 3) [4].

**Table 3:** AHA/ACC recommendations for classification of valve severity [56]

	Aortic sclerosis	Mild	Moderate	Severe
Aortic jet velocity (m/s)	≤2.5	2.6-2.9	3.0-4.0	>4.0
Mean gradient (mmHg)	–	<20	20-40	>40
AVA (cm^2^)	–	>1.5	1.0-1.5	<1.0

### Cine imaging and determination of aortic valve area

**Fig. 6:**
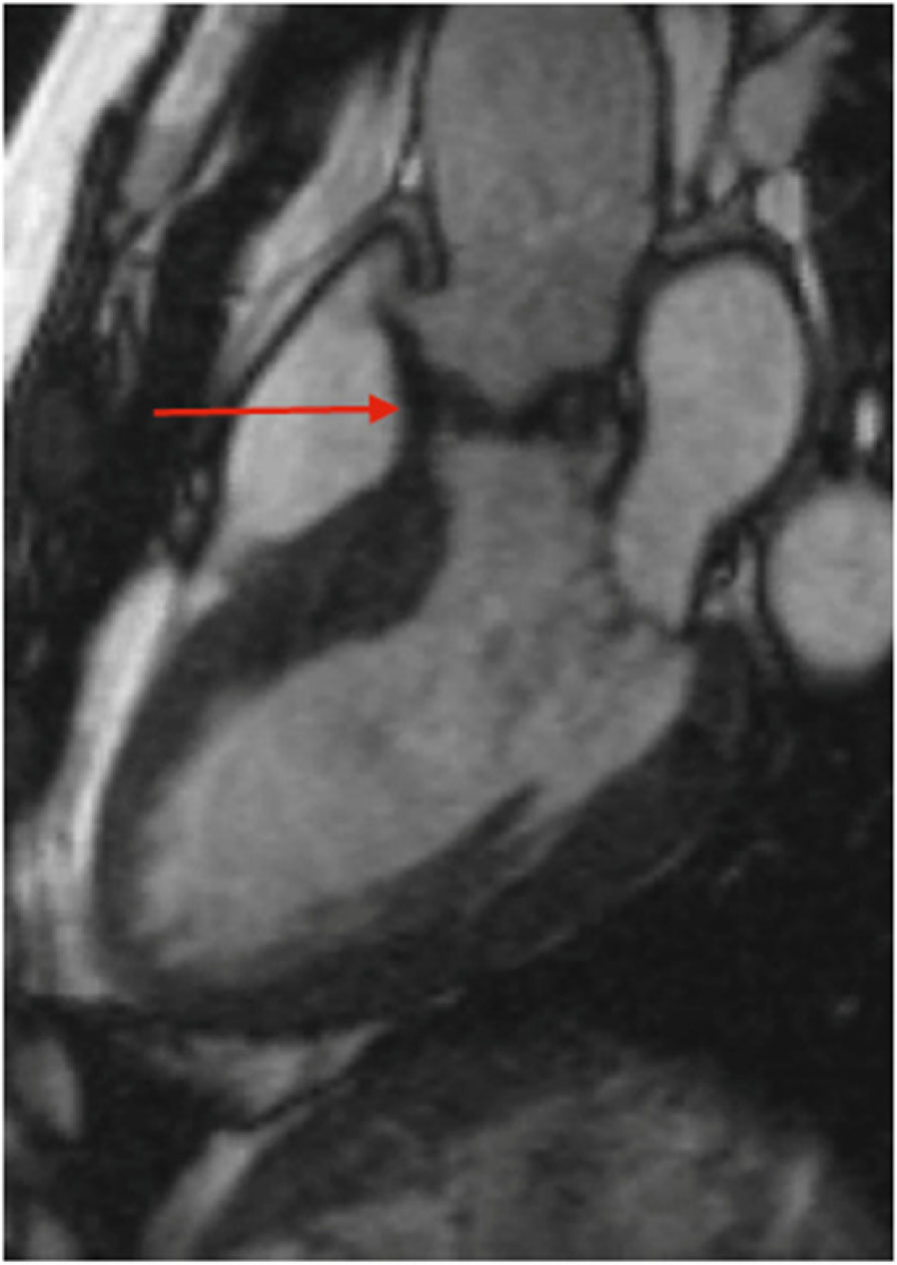
Three-chamber SSFP cine image of patient with calcific aortic stenosis. Calcification of the aortic valve (arrow) results in signal void on SSFP images. Source: University Hospitals of Leicester NHS Trust

CMR imaging in AS begins with standard three-chamber and coronal SSFP cine views, which provide a visual assessment of the aortic valve, LV, and LV outflow tract structure and function. All AS results in calcification of the aortic valve, which appears as signal void on CMR (Fig. 6).

**Fig. 7:**
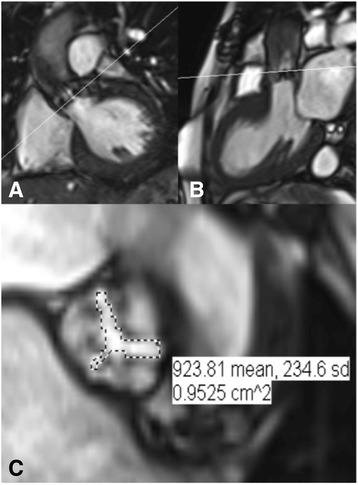
CMR-derived AV area by 2D planimetry. SSFP cine sequences of (**a**) the LV outflow tract and (**b**) three-chamber with LV outflow tract showing restricted AV leaflets and a stenotic jet. The perpendicular white lines indicate the slice position used for planimetry. (**c**) Direct planimetry of the AV orifice during systole. The calculated aortic valve area (0.95cm^2^ indicates severe AS). Source: University Hospitals of Leicester NHS Trust

More detailed aortic valve anatomy is achieved by through-plane SSFP imaging. Planimetry of the orifice should be precisely at the level of the valve tips, which is defined as the minimum area on any slice. Multiple, thin (4–5 mm) slices parallel to the valve should be acquired and we tend to use 0 slice gap, or even half moves of the positioning slice so that there is overlap (2–3 mm) between consecutive slices. This will enable direct planimetry of the valve orifice during systole (Fig. 7). This is the preferred method for grading severity. This method correlates well with aortic valve areas measured by cardiac catheterisation, trans-thoracic and trans-oesophageal echo, and direct measurement of autopsy specimens [17, 18]. Planimetry, however, can be suboptimal in cases of heavily calcified aortic valves due to signal void and stenotic jet turbulence [4]. A spoiled gradient echo pulse sequence can be used as an alternative and is recommended at 3 T as there is less flow artefact compared to SSFP [19].

### Quantification by flow mapping

Trans-valvular velocity is measured by velocity mapping, as described above. It is important that the correct slice position is identified for flow measurement, to maximise the accuracy of assessment. Initially in-plane velocity mapping in the LV outflow tract enables the identification of the area of maximal velocity, usually situated just beyond the valve tips in systole. Through-plane velocity mapping perpendicular to this identified area of maximal velocity is then performed, from which peak velocity is measured. Velocity encoding (VENC) is set manually on the phase contrast sequence and must be adjusted to avoid aliasing. Aliasing appears in the centre of the flow area, as the opposite colour to forward flow. We tend to gauge the required VENC based on how stenotic the valve appears on cine imaging and usually a value of 2.5-4 m/s is chosen. Although some vendors have built-in adjustments of VENC for aliasing, the sequence should be repeated with a higher VENC if there is significant aliasing (>1-2 pixels). Any velocity > 4 m/s should be considered as severe AS unless there is severe combined aortic regurgitations (AR), which increases stroke volume and flow across the valve.

Peak velocity in AS measured by CMR has been validated against continuous-wave Doppler echocardiography and there is a tendency for CMR to underestimate peak velocity [4]. This is the result of partial-volume effects within the vena contracta of very high velocity jets, as well as artefacts generated from turbulent jets as already described [3, 4]. However, CMR is advantageous in cases where correct echo beam alignment through the stenotic jet is difficult.

### Aorta imaging

Another benefit of CMR is the ability to characterise the aortic anatomy, which may be affected by post-stenotic aortic root dilatation, and can influence subsequent surgical management. This is especially important in those with a bicuspid aortic valve, where there is particular susceptibility to aortic root dilatation and an association with aortic coarctation. Recent 4D flow studies have suggested that abnormal helical and chaotic flow patterns associated with bicuspid aortic valve disease are likely to be the cause of aortic dilatation, rather than an inherited aortopathy [20, 21].

In patients being considered for aortic valve intervention it is essential that aortic annulus measurements are given. This helps guide size of prosthesis and is particularly important for transcutaneous valve insertion [22].

### Tissue characterisation

Tissue characterisation by LGE enables the identification of replacement fibrosis in subjects with AS. Over one quarter of patients with AS demonstrate areas of LGE, which correlates with disease severity and is an independent predictor of mortality [23, 24]. When LGE is evident in AS, the typical pattern is one of a patchy mid-wall distribution. The regions of hyperenhancement represent areas of focal fibrosis, but more diffuse myocardial fibrosis may be underestimated by LGE [25]. Markers of diffuse myocardial fibrosis, such as extracellular volume fraction (ECV) and native T1 relaxation times, are also increased in AS and progress with disease severity [26, 27]. Several studies are currently in progress assessing whether multiparametric CMR can predict symptom development and recently, indexed ECV has been shown to be more strongly associated with mortality than LV mass index in AS [28].

### Aortic regurgitation (AR)

Several disease processes can lead to the development of aortic regurgitation (AR). The commonest of these are degenerative and bicuspid aortic valves, although endocarditis of the aortic valve or diseases of the aortic root causing functional dilatation (e.g. hypertension, aortic dissection and Marfan syndrome) may also cause AR [29]. CMR evaluation of AR is advantageous owing to the high degree of accuracy for assessment of LV volumes and function as well as the capability for determining aortic regurgitant volumes [3, 29]. As with AS, periodic monitoring of AR is recommended [30]. Echocardiographic guidelines for grading the severity of AR are shown in Table 4.

**Table 4:** ESC guidelines on grading the severity of AR by echocardiography [57]

	Mild	Moderate	Severe
Qualitative			
Aortic valve morphology	Normal/abnormal	Normal/abnormal	Abnormal/flail/large coaptation defect.
Colour flow AR jet width	Small in central jets	Intermediate	Large in central jets, variable in eccentric jets
Continuous wave signal of AR jet	Incomplete/faint	Dense	Dense
Diastolic flow reversal in descending aorta	Brief, protodiastolic flow reversal	Intermediate	Holodiastolic flow reversal (end-diastolic velocity > 20 cm/s)
Semi-quantitative			
Vena contracta width (mm)	<3	Intermediate	>6
Pressure half-time (ms)	>500	Intermediate	<200
Quantitative			
Effective regurgitant orifice area (mm^2^)	<10	10-29	≥30
Regurgitant volume (mL)	<30	30-59	≥60

### Cine imaging

**Fig. 8:**
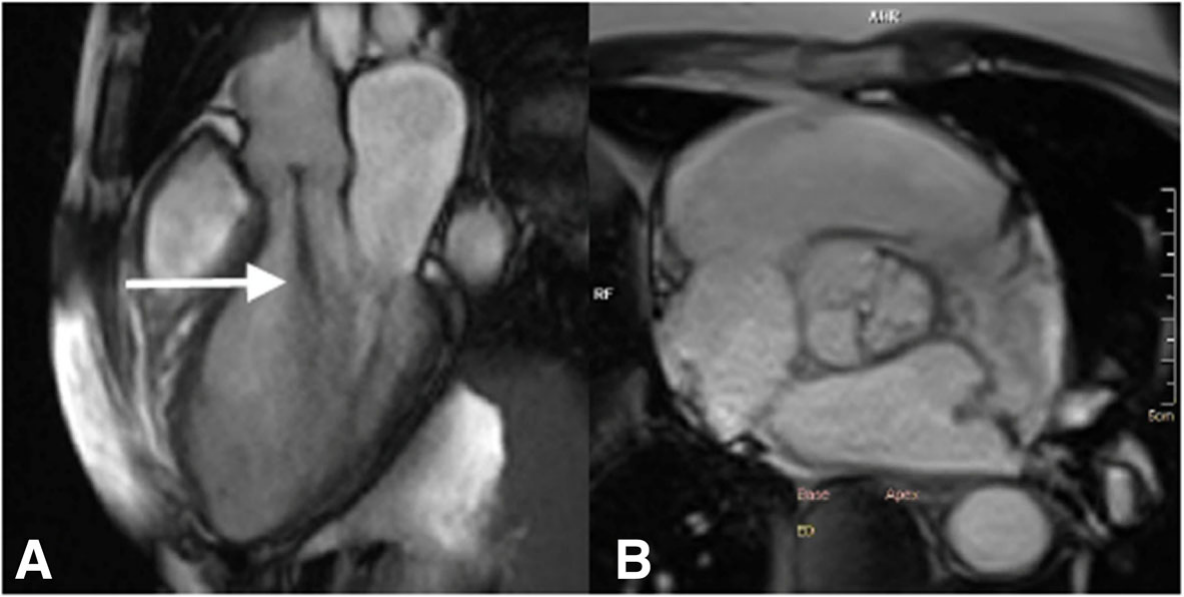
SSFP cine three-chamber image showing aortic regurgitation. **a** The central regurgitant jet (arrow) is visible as signal void on SSFP sequences. **b** Visualisation of the central AV regurgitant orifice by direct planimetry. Source: University Hospitals of Leicester NHS Trust

A similar approach to AS is used for CMR imaging in AR. This begins with the standard visual assessment of the aortic valve, LV, and LV outflow tract structure and function using SSFP cine sequences (Fig. 8a). The impact of AR on the LV is assessed with accurate LV volume and function quantification by CMR. Serial measurements can be performed with high reproducibility and provide useful information regarding disease progression. In a multi-centre observational study LV EDV >246 mL predicted those patients who developed a class I guideline indication for surgery [31].

Valve morphology (e.g. bicuspid/tricuspid) and aortic root anatomy are of particular interest in patients with AR. Planimetry of the valve orifice should be acquired as for AS (Fig. 5b). The regurgitant orifice area may be measured directly by planimetry. It should be noted that calculation of the regurgitant jet area or length are not reliable indices of disease severity and are therefore not usually performed [4]. Assessment of the aortic root anatomy can aid in the identification of the cause of AR as well as determining the requirement for aortic root repair/replacement alongside AVR.

### Calculation of AR severity

**Fig. 9:**
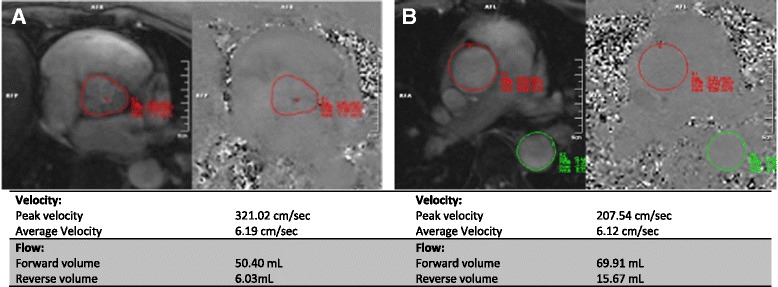
Phase contrast velocity mapping in aortic regurgitation. Aortic flow has been measured at (**a**) aortic level and (**b**) pulmonary artery level. Note the difference in forward and reverse volumes generated by velocity mapping at the different sites. At (**a**) the aortic level the regurgitant fraction is 12% compared with 23% at (**b**) the PA level, which reflects lower calculated stroke volumes if flow is calculated at aortic level. Source: University Hospitals of Leicester NHS Trust

Phase-contrast velocity mapping is used to calculate forward and reverse flow per cardiac cycle. Positioning of the imaging slice is important to ensure accurate assessment [32]. In AR the imaging slice is usually positioned at the level of sinotubular junction allowing direct measurement of the trans-valvular forward and regurgitant volumes. However, others advocate measurement at the valve tips or annulus and a recent paper in normal subjects showed that stroke volume measured at the annulus was more closely associated with LV stroke volume than flow measured at the sino-tubular junction. We have seen multiple cases where the stroke volume was lower at the annulus and we suggest that flow is measured at the annulus, sino-tubular junction and pulmonary artery bifurcation (Fig. 9) [33].

For inexperienced centres, we recommend that aorta flow is routinely measured in patients between contrast administration and late gadolinium enhanced image acquisition. This allows centres to obtain a good ‘feel’ for how accurate aorta flow measurement is against LV stroke volume.

Regurgitant volume is simply the difference between LV stroke volume from the LV measurements and forward aortic volume. From these the regurgitant fraction is calculated (regurgitant volume/forward volume × 100) [3]. Although there is a moderate correlation between regurgitant volumes measured by echocardiography and CMR, the limits of agreement are wide but observer variability is significantly lower with CMR, suggesting this may be the preferred method of assessment [34]. Flow mapping alone can be used to calculate severity of AR and flow reversal can be directly quantified with this technique. Alternatively the regurgitant volume can be calculated by cine assessment and comparison of LV and right ventricle (RV) stroke volumes. The difference in ventricular stroke volumes represents the regurgitant volume, assuming no other valve disease is present [3, 4].

Regurgitant fraction is an independent predictor of outcome in patients with AR, and a regurgitant fraction of >33% has been shown to predict the likelihood of requiring surgery within nine years [31]. A combination of LV EDV and regurgitant fraction are proposed as powerful discriminators for the likelihood to progression to surgery but randomised trials are required to demonstrate clinical benefit [31].

### Mitral stenosis (MS)

Rheumatic heart disease is by far the commonest cause of MS, accounting for over 95% of cases [35]. As a consequence the prevalence of MS has fallen with the decline of rheumatic fever in the developed world [36]. Rarer causes include carcinoid, Fabry’s disease, mucopolysaccharidoses, Whipple’s disease, rheumatoid arthritis, gout and congenital mitral stenosis [37]. Over one-third of patients with rheumatic mitral valve disease have involvement of other valves, most commonly the aortic valve [35]. This should be considered as part of the CMR evaluation of MS. Severity of MS by echo is graded predominantly by valve area, although mean gradient and pulmonary artery pressure are also useful markers of severity (Table 5) [38].

**Table 5:** Echocardiographic parameters for determining the severity of MS [38]

	Mild	Moderate	Severe
Valve area (cm^2^)	>1.5	1.0–1.5	<1.0
Mean gradient (mmHg)	<5	5-10	>10
Pulmonary artery pressure (mmHg)	<30	30-50	>50

**Fig. 10:**
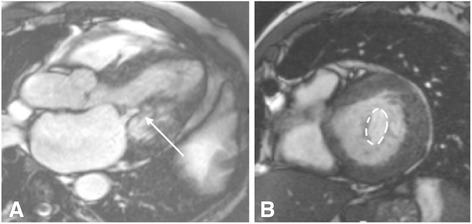
CMR evaluation of MS. **a** SSFP cine images enable visualisation of the stenotic jet in MS (arrow). **b** Planimetry of the mitral valve (dashed line) to calculate valve orifice area should be performed in diastole. Source: University Hospitals of Leicester NHS Trust

Standard assessment of MS begins with the standard SSFP cine views, followed by mitral valve (MV) planimetry and MV flow velocities. The two-chamber, four-chamber and LVOT views are of particular interest initially, with visualisation of the signal void generated by the stenotic jet (Fig. 10a). The impact of MS on the left atrium is easily quantified by these images. MV planimetry may be performed in a manner similar to the AV (Fig. 10b). An imaging plane close to the MV tips should be acquired during diastole. Multiple, thin (4-5 mm) slices should be imaged parallel to the mitral annulus to ensure accuracy.

**Fig. 11:**
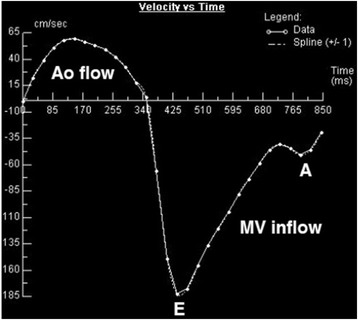
Flow-time curve generated by CMR phase-contrast velocity mapping across the mitral valve (MV) in a patient with MS. Aortic (Ao) and mitral valve (MV) flow are shown. E and A waves are labelled at their respective peaks. The E deceleration time is prolonged, indicating mitral stenosis. Source: University Hospitals of Leicester NHS Trust

Mitral diastolic inflow velocities performed with Doppler echocardiography correlate well with CMR-derived values [39, 40]. Mitral flow velocities curves are acquired by phase-contrast velocity mapping in the same plane as used for planimetry of the MV (Fig. 11). In patients with atrial fibrillation, however, the accuracy of flow measurements by CMR is limited [41].

### Mitral regurgitation (MR)

MR is broken down in to primary and secondary causes. In primary MR there is disease affecting the mitral valve (MV) leaflets or the MV apparatus. In secondary (or functional) MR, the MV is normal and regurgitation results from annular or ventricular dilatation, which causes reduced or absent leaflet coaptation [42]. In patients with severe MR undergoing surgical intervention, the commonest causes are mitral valve prolapse, ischaemic MR, rheumatic heart disease and endocarditis [43]. Guidelines for grading severity of MR are shown in Table 6.

**Table 6:** ACC/AHA guidelines for grading severity of MR [30]

	Mild	Moderate	Severe
Qualitative			
Angiographic grade	1+	2+	3-4+
Colour Doppler jet area	Small, central jet (<4cm^2^ or <20% LA area)	Signs of MR greater than mild present, but no criteria for severe MR.	Vena contracta width greater than 0.7 cm with large central MR jet (area < 40% of LA area) or with a wall-impinging jet of any size, swirling in LA.
Doppler vena contracta width (cm)	<0.3	0.3-0.69	≥0.7
Quantitative (cardiac catheterisation or echo)			
Regurgitant volume (ml)	<30	30-59	≥60
Regurgitant fraction (%)	<30	30-49	≥50
Regurgitant orifice area (cm^2^)	<0.2	0.2-0.39	≥0.4
Additional essential criteria			
LA size			Enlarged
LV size			Enlarged

The unlimited imaging planes available through CMR are advantageous for evaluation of the complex MV anatomy. Quantification of regurgitation alongside LV volumes and function remain the major benefits of CMR in the assessment of MR. A visual assessment of the signal void created by the regurgitant jet can give an initial impression of the severity of MR, with wider jets indicative of more severe disease, particularly in the presence of a core area of high signal within the jet. However quantification should be carried out as described below.

### Cine imaging in MR

**Fig. 12:**
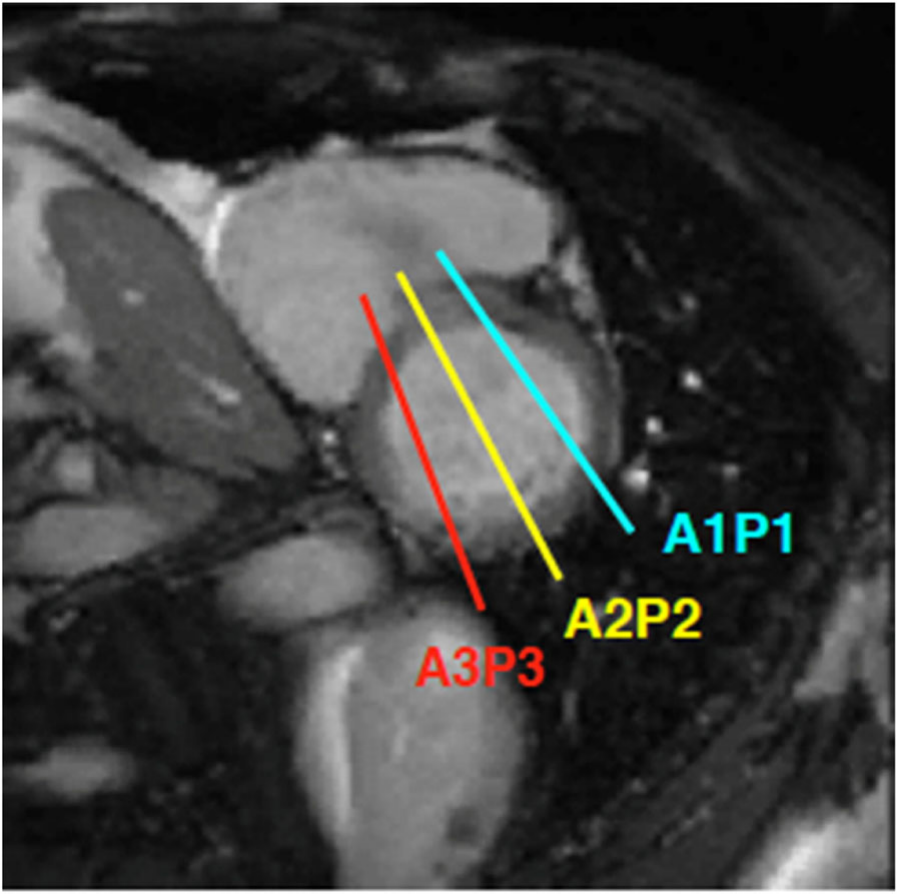
Suggested imaging planes for visualisation of MV leaflets, according to the coaptation of the individual scallops. Slice position is planned from the basal short axis image (bottom left panel). Cine sequences are then acquired perpendicular to the mitral commissure corresponding to each of the three scallops, denoted by the coloured lines and respective coloured text. Source: University Hospitals of Leicester NHS Trust

A useful approach to assessment of the MV leaflet anatomy is to image the valve in three planes according to the coaptation of the individual scallops (i.e. A1P1, A2P2 and A3P3) (Fig. 12). This will enable the identification of the site of regurgitation or prolapse. A basal short axis slice through the MV commissure can be used to plan subsequent imaging planes. SSFP cine image slices through each of the three scallops can then be acquired. Direct planimetry though the mitral annulus may also be performed to enable measurement of the regurgitant orifice area [3]. A regurgitant orifice of ≥40mm^2^ portends a poor prognosis in patients with MR and has been proposed as an indication for surgical intervention [44]. Alternative imaging can be performed by a stack of axial slices or perpendicular to the four-chamber view.

### Quantification of MR

**Fig. 13:**
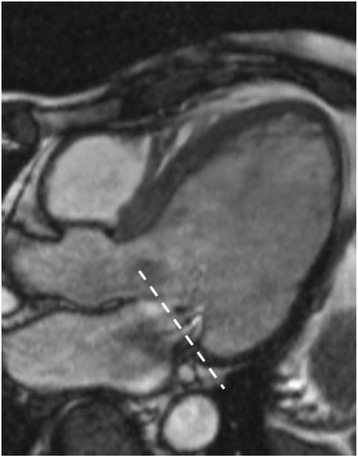
Three-chamber SSFP cine image in subject with ischaemic MR. Dashed line indicates the slice position used for through-plane phase-contrast velocity mapping. Care should be taken that this is located perpendicular to the regurgitant jet. Source: University Hospitals of Leicester NHS Trust

Two methods for quantification of MR are possible by CMR: direct and indirect. The preferred method is indirect quantification, which enables calculation of MR volume by subtracting aortic forward flow (by phase-velocity mapping, as described above, or pulmonary flow) from the LV stroke volume. MR regurgitant fraction is calculated similar to that for the aortic valve: regurgitant volume/LV stroke volume × 100. Alternatively the difference in stroke volume between the LV and RV can be used to calculate the MR volume, although this is based on the assumption that no other valve lesion is present. For direct quantification of MR, through-plane phase-contrast velocity mapping may be undertaken, with care being taken to ensure the imaging plane is perpendicular to the regurgitant jet on the atrial side of the valve (Fig. 13). Direct quantification is made challenging by the highly mobile nature of the MV and the often eccentric jets of MR [3]. Nevertheless there is good agreement for MR quantification between the direct and indirect methods, although the indirect method is preferred in patients with variable heart rates where phase-contrast mapping may be prone to errors [41].

Indirect quantification of MR by CMR correlates only modestly with echocardiography but has markedly lower inter- and intra-observer variability [34, 45]. Moreover, emerging data show that there is marked discrepancy in the severity of MR assessment using echocardiography and CMR [46]. Whilst these findings do not prove that CMR is superior to echo, the fact that reductions in LV volumes following MV surgery were closely related to regurgitant volumes measured on CMR but not echocardiography do suggest that CMR is preferred method of quantification [46].

A regurgitant fraction of ≥40% is proposed as the threshold for surgery in asymptomatic MR [47]. However, there are no published data on cut-offs to predict outcome using CMR and there are no randomised trials comparing management of patients using CMR and echocardiography, so caution must be exercised in interpreting values in the meantime.

### Right-sided heart valve disease

CMR is the gold-standard tool for quantification of RV volumes and function. Furthermore, CMR enables imaging of the pulmonary valve and RV outflow tract with precision not possible with 2D echo. The complex structure of the RV makes volumetric assessment by echo difficult. CMR is therefore the preferred modality for RV and pulmonary valve disease evaluation.

### Pulmonary valve disease

**Fig. 14:**
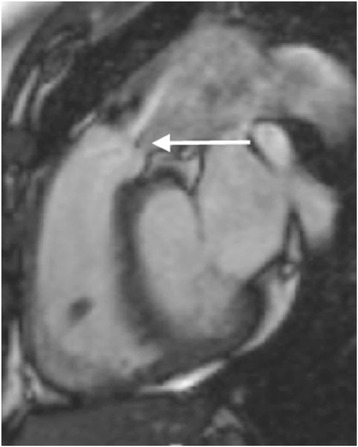
SSFP RV outflow tract cine image in a subject with PS. Arrow indicates the location of the pulmonary valve, after which the stenotic jet is seen. Source: University Hospitals of Leicester NHS Trust

Phase-contrast velocity mapping for quantification of pulmonary regurgitation (PR) has been validated against CMR derived LV and RV stroke volumes [48]. Assessment of PR by echo is only qualitative and comparison with CMR is therefore limited. As with AR, flow mapping alone can be used to calculate severity of PR and flow reversal can be directly quantified with this technique. Trivial PR is a common finding in normal subjects [49]. The occurrence of clinically significant PR is primarily the result of congenital cardiac disease – usually following surgical repair of tetralogy of Fallot [50]. Detailed CMR assessment of PR is therefore beyond the scope of this review. Pulmonary stenosis (PS) is easily visualised using CMR by acquiring SSFP cine views of the RV outflow tract (Fig. 14).

### Tricuspid valve disease

**Fig. 15:**
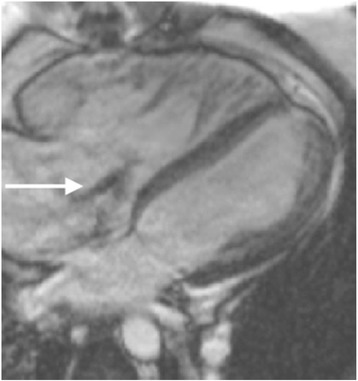
SSFP four-chamber cine image in a subject with TR. The regurgitant jet (arrow) is seen as signal void on SSFP cine images. Source: University Hospitals of Leicester NHS Trust

Assessment of the tricuspid valve (TV) by CMR is performed in a manner similar to that of the MV, and axial cuts may be particularly useful. Tricuspid regurgitation (TR) is seen as signal void on SSFP cine images, best seen in the long axis slices (Fig. 15). For quantification of TR, an indirect method is preferred. Through-plane phase-contrast velocity mapping in the pulmonary artery is undertaken to calculate forward flow volumes. This is subtracted from the total RV stroke volume to provide a TR regurgitant volume [4]. Tricuspid stenosis is rare and not ordinarily assessed by CMR [3].

### Prosthetic heart valves

CMR is a safe technique for imaging patients with prosthetic heart valves. All prosthetic valves can be imaged safely at a field strength of 1.5 T and the vast majority at 3 T, although not all prostheses have been tested at 3 T [51]. In the event of concern the safety of any prosthesis can be checked using online resources.

**Fig. 16:**
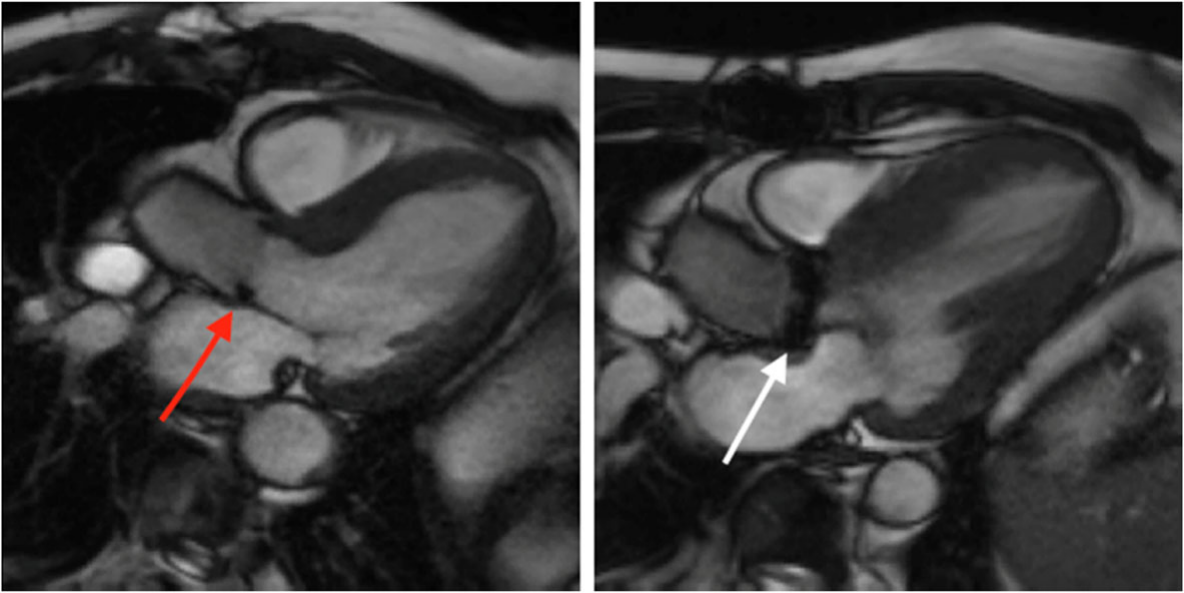
SSFP three-chamber cine images in two subjects with prosthetic aortic valves. The degree of artefact is determined by the proportion of metal in the valve prosthesis. The red arrow points to a patient with a bioprosthetic AVR, with minimal artefact generated by the small metal component of the supporting structure of the valve. By comparison, a much greater degree of artefact is created by the metallic AVR (white arrow), which has larger metallic component. Source: University Hospitals of Leicester NHS Trust

The primary issue when imaging prosthetic valves is the occurrence of artefacts. SSFP cine sequences are highly susceptible to artefacts produced by ferromagnetic objects. The proportion of metal within the prosthetic valve therefore determines the degree of artefact (Fig. 16). This can vary from very little to large areas of artefact that obscure the surrounding anatomy [52, 53]. In the event of very severe artefacts with SSFP sequences, a spoiled gradient echo sequence can be used to acquire cine images. Gradient echo is less susceptible to artefacts from prosthetic valves, but this is at the expense of lower signal to noise [53].

Through-plane velocity mapping can be applied to prosthetic heart valves in the same way as for native valves. Varying degrees of signal voids occur above and below prosthetic valves. The imaging plane should be positioned downstream of the signal void artefact [3, 53]. Different valve types generate different flow patterns on phase-contrast imaging [3].

Finally, CMR is extremely valuable for assessing reverse ventricular remodelling following valve interventions, particularly when comparing different valve types as small numbers of patients are required to show statistically significant differences [54, 55].

## Conclusions

CMR can be used for the comprehensive evaluation of VHD. The main strength of CMR lies in the highly accurate and reproducible assessment of ventricular volumes and function in patients with left- or right-sided valve disease. The ability to image in unlimited planes is particularly important in patients with right-sided valve disease, which is poorly evaluated by echo. The quantification of regurgitant volumes/fraction with CMR is a particularly promising area, and one which is established for pulmonary regurgitation following surgical correction of Fallot’s. However further prospective studies, and ideally randomised controlled trials comparing MR and echocardiography, are required before the assessment of left sided valve lesions can be considered the clinical routine. The limitations of CMR should be borne in mind and CMR can never replace echo for use at the bedside or in the critically ill patient. Despite these shortcomings CMR is an exciting non-invasive imaging modality in patients with VHD and improvements in techniques and technologies are likely to enhance its utility in clinical practice.

## Abbreviations

ACC: American College of Cardiology
AHA: American Heart Association
AR: Aortic regurgitation
AS: Aortic stenosis
CMR: Cardiac magnetic resonance imaging
ECV: Extracellular volume fraction
EDV: End-diastolic volume
EF: Ejection fraction
ESV: End-systolic volume
LGE: Late gadolinium enhancement
LV: Left ventricle
LVM: Left-ventricular mass
MR: Mitral regurgitation
MS: Mitral stenosis
MV: Mitral valve
PR: Pulmonary regurgitation
RV: Right ventricle
SSFP: Steady state free precession
SV: Stroke volume
TV: Tricuspid valve
VENC: Velocity encoding
VHD: Valvular heart disease
